# Incomplete Atypical Femoral Fracture With Severe Bowing Treated by Intramedullary Nail and Corrective Osteotomy

**DOI:** 10.7759/cureus.38175

**Published:** 2023-04-26

**Authors:** Shinsuke Sato, Daisuke Kitamura, Shuhei Murase, Yuji Tanaka, Kiyofumi Yamakawa

**Affiliations:** 1 Department of Orthopaedics, Tokyo Metropolitan Bokutoh Hospital, Tokyo, JPN

**Keywords:** intramedullary nail, corrective osteotomy, surgical technique guide, trauma, incomplete atypical femoral fracture

## Abstract

We present the case of an 82-year-old female who had difficulty walking due to right thigh pain caused by incomplete atypical femoral fracture (AFF). The femoral bowing was so severe that intramedullary nail insertion was impossible, so we performed a corrective osteotomy of the femur and inserted the intramedullary nail. Postoperatively, the femoral pain disappeared, and bone fusion was achieved at one year and two months postoperatively. In cases of incomplete AFF with very severe femoral bowing, internal fixation with an intramedullary nail combined with corrective osteotomy of the femur is useful.

## Introduction

Atypical femoral fracture (AFF) is a generic term for femoral fractures that occur as a result of nontraumatic or minor trauma, usually after long-term use of bisphosphonates. Incomplete AFFs are characterized by a thickened image confined to the lateral cortex and an incomplete fracture line and may have prodromal symptoms such as dull pain in the groin or thigh but often remain asymptomatic and only become symptomatic when they become a complete fracture and the patient is unable to walk. Although prophylactic fixation is recommended for the treatment of symptomatic incomplete AFF, some reports recommend internal fixation to prevent complete fracture, control the pain, and improve activities of daily living (ADLs) [1,2]. In these cases, however, the bowing is so severe that an intramedullary nail cannot be inserted. We performed internal fixation with the intramedullary nail combined with corrective osteotomy for incomplete AFF with very severe femoral bowing. We report the clinical course of this case. As far as we could find, no similar cases have been published.

The patient was informed that the data of the case would be submitted for publication, and she gave her consent.

## Case presentation

**Figure 1:**
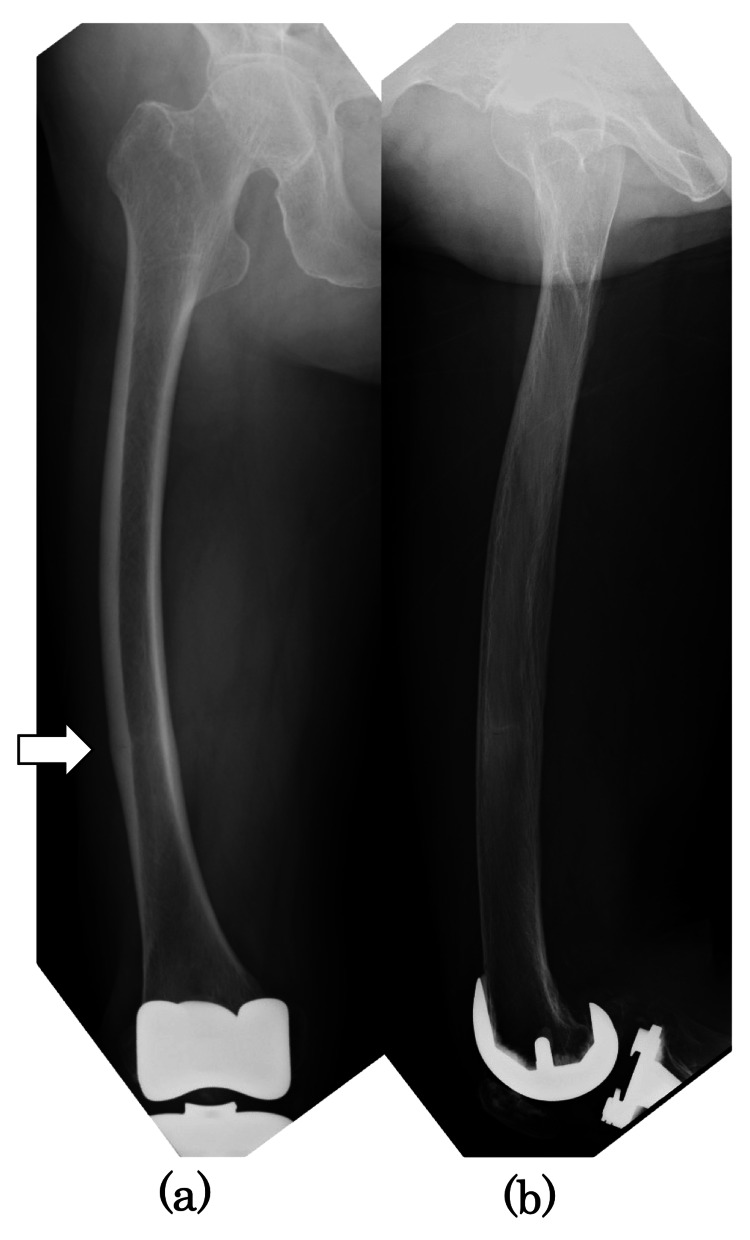
Preoperative radiographs Preoperative radiographs: (a) anteroposterior and (b) lateral images. The white arrow showed a thickening of the lateral cortical bone in the diaphysis.

An 82-year-old female with no history of bisphosphonate use and a history of right total knee arthroplasty (TKA) for osteoarthritis 7.5 years earlier presented to our hospital with a chief complaint of right distal lateral thigh pain. One month before the presentation, the pain had developed without a history of falls or trauma, and she had difficulty walking. Radiographs showed periosteal beak-like thickening of the lateral cortex (Figure 1).

**Figure 2:**
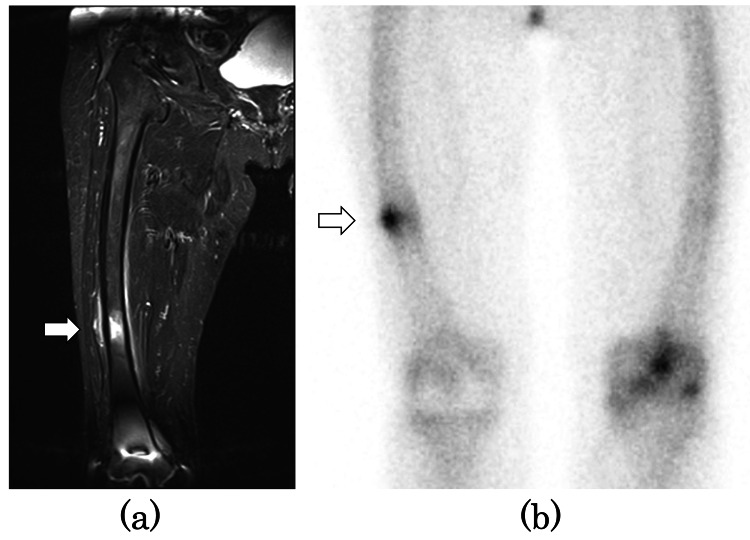
Preoperative MRI STIR coronal image and bone scintigraphy (a) MRI STIR coronal image showed bone marrow edema in the diaphysis of the right femur (white arrow). (b) Bone scintigraphy showed a stress fracture of the right femur (white arrow). MRI: magnetic resonance imaging, STIR: short tau inversion recovery

Magnetic resonance imaging (MRI) short tau inversion recovery (STIR) showed a high signal (Figure 2a), and bone scintigraphy showed uptake (Figure 2b). We diagnosed it as incomplete AFF.

**Figure 3:**
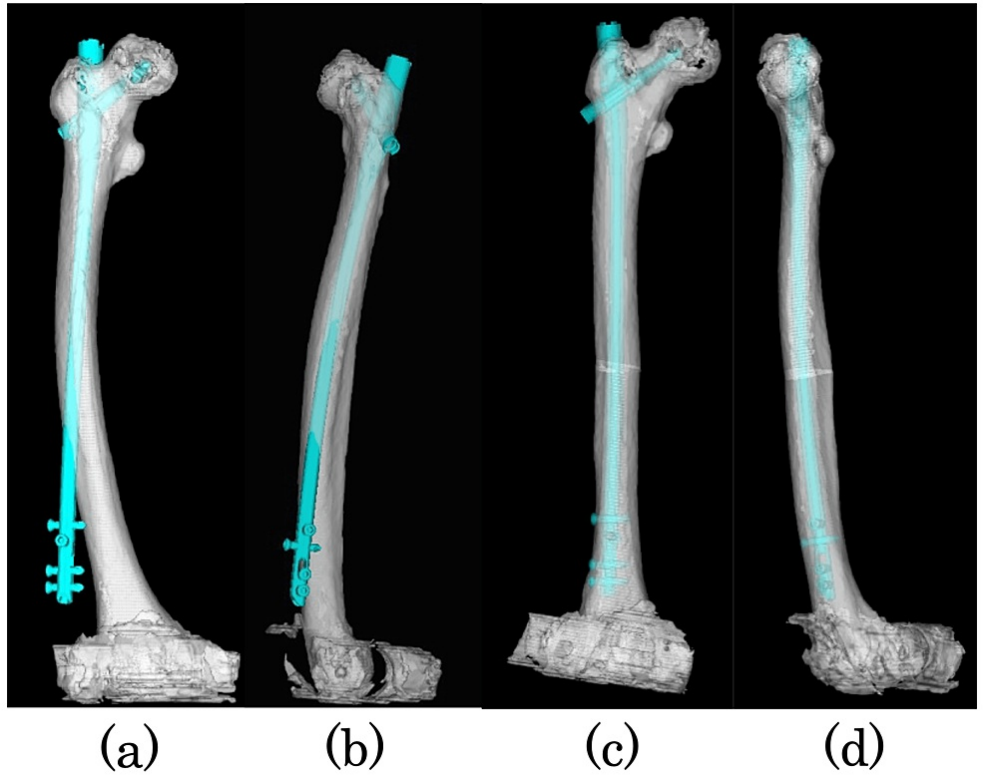
Preoperative 3D templates Preoperative 3D templates: (a) anteroposterior and (b) lateral images. They showed severe femoral bowing and the impossibility of intramedullary nailing insertion. Preoperative 3D templates after corrective osteotomy: (c) anteroposterior and (d) lateral images. The intramedullary nailing can be inserted into the femur. 3D: three dimensional

Internal fixation with the intramedullary nail was planned before the complete fracture, but in the preoperative planning, nail insertion was impossible due to the severe femoral bowing (Figure 3), and we decided to perform a corrective osteotomy of the femur. The corrective osteotomy site (the distance from the osteotomy to the fracture site and greater trochanter), corrective angle, and nail placement were planned using 3D template software (ZedTrauma, Lexi, Tokyo, Japan) and 2D drawings.

**Figure 4:**
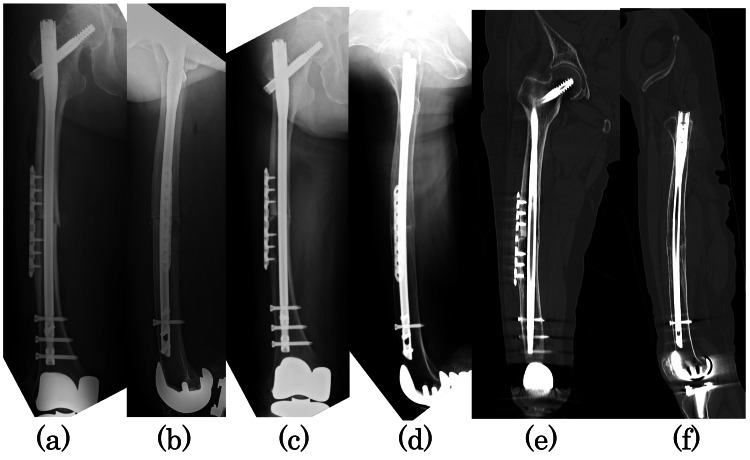
Radiographs taken immediately after surgery and at one year and two months postoperatively Immediate postoperative radiographs: (a) anteroposterior and (b) lateral images. Radiographs at one year and two months postoperatively: (c) anteroposterior and (d) lateral images. Plain computed tomography at one year and two months postoperatively: (e) coronal and (f) sagittal images. Anterior, posterior, and medial bony fusion of the osteotomy has been achieved, and bony bridging has been achieved on the lateral side.

The patient was operated on in the left lateral decubitus position, and the fracture was identified and marked under fluoroscopy. The previously measured distance from the fracture site to the osteotomy site was used to identify the osteotomy site, and the approach was made from the lateral femur centered on the osteotomy site. Closed wedge osteotomy was performed using an air tome and chisel at the angle planned preoperatively. The nail (CM long nail, Zimmer Biomet, Warsaw, IN, USA) was inserted through the pre-planned insertion site of the greater trochanter. The locking plate (LCP small plate, DePuy Synthes, Raynham, MA, USA) was placed on the lateral side of the fracture site due to the large movement caused by rotation. Manual compression was applied to the fracture site, and autogenous bone from the osteotomy was grafted to the fracture site (Figure 4a, 4b).

No weight-bearing was allowed for two weeks postoperatively, partial weight-bearing up to 50% was allowed from two weeks postoperatively, and full weight-bearing was allowed from six weeks postoperatively. Teriparatide and low-intensity pulsed ultrasound (LIPUS) were started immediately after surgery. Preoperative femoral pain disappeared, and bone fusion was achieved in 14 months (Figure 4c, 4d, 4e, 4f).

## Discussion

**Figure 5:**
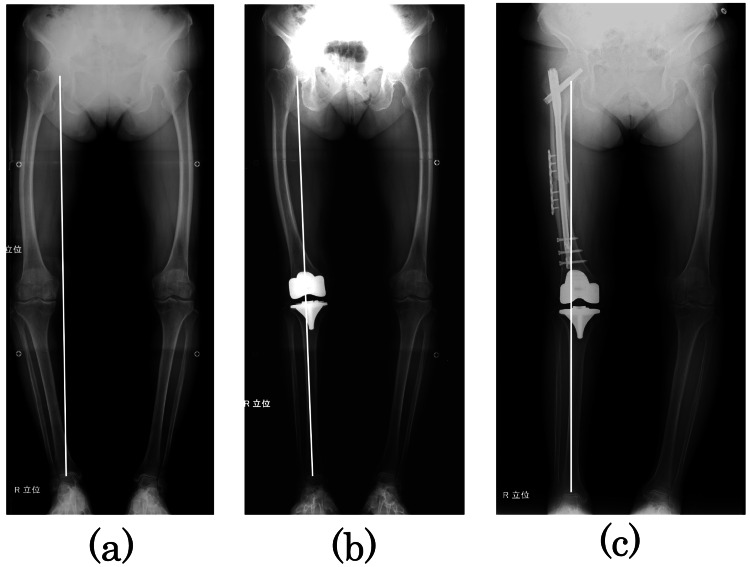
Radiographs of the entire length of the lower limb Radiographs of the entire length of the lower limb: (a) immediately before TKA surgery, (b) immediately after TKA surgery, and (c) immediately after this surgery. The white line shows the Mikulicz line. TKA: total knee arthroplasty

Although AFF has been reported as a complication of long-term bisphosphonate use [3], the 2010 American Society for Bone and Mineral Research (ASBMR) Task Force case definition of AFFs does not necessarily require bisphosphonate use, and bisphosphonate use is considered a minor feature [4]. Sasaki et al. [5] have reported that AFF is caused by stress concentration due to femoral bowing. This patient had no history of bisphosphonate. The likely cause of the AFF was stress concentration caused by severe bowing. In addition, the change in alignment after the right TKA could have accelerated the stress concentration. The Mikulicz line passes through the medial side of the knee joint (Figure 5a), but the tibia follows the Mikulicz line, and only the femur passes outside the Mikulicz line after TKA (Figure 5b). In other words, before TKA, both the femur and tibia were stressed, but after TKA, only the femur was stressed, which may have led to AFF. Complementing this, there was no evidence of AFF in the left femur showing similar bowing.

There is no consensus on the optimal timing of surgery for incomplete AFF. It has been reported that surgery results in faster bone fusion than conservative treatment [6-8]. Blood et al. [2] recommended surgery if there is radiographic evidence of AFF and pain. Min et al. [9] developed the scoring system to predict the risk of complete fracture in incomplete AFF and reported that prophylactic internal fixation should be performed if the total score is 8 or more, based on a score of 1-3 points each for the following four items: fracture height, pain intensity, contralateral status, and degree of the insufficiency fracture line. In this case, 2 points were given for fracture height, 3 points for pain intensity, 2 points for contralateral status, and 1 point for the degree of the insufficiency fracture line, for a total of 8 points. Our choice of surgical treatment in this case seems reasonable.

The standard surgical treatment for incomplete AFF is internal fixation with an intramedullary nail as for complete AFF [10]. However, there are cases where the bowing is so severe that the procedure is difficult. In such cases, there is an option to wait until the complete fracture, but it will force patients to deal with the pain that limits ADLs and the pain that occurs with a complete fracture.

Several surgical techniques for AFF with severe bowing have been reported. The following methods have been reported: insertion of a straight nail through the apex of the greater trochanter [11], insertions of a nail with external rotation [11], insertions of a contralateral Zimmer Biomet Natural nail [12], addition of the lateral plate [13], and use of the elastic intramedullary nail [14]. In this case, intramedullary nail alone was not possible with either method, so we planned the corrective osteotomy. The corrective osteotomy required detailed preoperative planning, and the 3D template software and 2D drawings were very useful.

## Conclusions

This case report describes a patient with incomplete AFF whose femoral bowing was so severe that a conventional intramedullary nail could not be inserted. We treated it with an intramedullary nail combined with a corrective osteotomy. Corrective osteotomies allow us to insert an intramedullary nail into the severely bowed femur. Corrective osteotomy can be considered in the surgical management of severe bowing AFF following appropriate preoperative planning.
